# Subsoiling practices change root distribution and increase post-anthesis dry matter accumulation and yield in summer maize

**DOI:** 10.1371/journal.pone.0174952

**Published:** 2017-04-06

**Authors:** Xuefang Sun, Zaisong Ding, Xinbing Wang, Haipeng Hou, Baoyuan Zhou, Yang Yue, Wei Ma, Junzhu Ge, Zhimin Wang, Ming Zhao

**Affiliations:** 1 Institute of Crop Sciences, Chinese Academy of Agricultural Sciences / Key Laboratory of Crop Ecophysiology and Cultivation, Ministry of Agriculture, Beijing, China; 2 College of Agronomy and Biotechnology, China Agricultural University, Beijing, China; Agroecological Institute, CHINA

## Abstract

Subsoiling is an important management practice for improving maize yield, especially for maize planted at high plant density. However, the affected physiological processes have yet to be specifically identified. In this study, field experiments with two soil tillage (CK: no-tillage, SS: subsoiling) and three planting densities (low: 45000 plants ha^−1^, medium: 67500plants ha^−1^, and high: 90000 plants ha^−1^) were conducted from 2010 to 2012 at Xinxiang, Henan province. Yield, canopy function, and root system were investigated to determine the associated physiological processes for improving maize production affected by soil tillage and plant density. Subsoiling significantly increased the grain yield of the low-, medium-, and high-planting densities by 6.21%, 8.92%, and 10.09%, respectively. Yield increase in the SS plots was mainly attributed to greater post-anthesis DMA and improved grain filling compared to CK plots. Greater green leaf area, leaf net photosynthetic rate, F_V_/F_m_ and ΦPSII in the SS plots were mainly contributed to enhanced dry matter production post-anthesis. This is mainly because subsoiling increased density of root dry weight in deep soil and root bleeding sap amount due to decreased the bulk density of the 0–30 cm soil profile layer. Density of root dry weight at 10–50 cm depth with SS increased by 40.68%, 32.17%, and 20.14% at low, medium, and high planting densities compared to CK, respectively, while the root bleeding sap amount increased by 17.41%, 15.82%, and 20.91%. These results indicate that subsoiling could change the root distribution and improve soil layer environment for root growth, thus maintaining a higher canopy photosynthetic capacity post-anthesis and in turn promoting DMA and yield, particularly at higher planting densities.

## Introduction

The Huang-Huai-Hai Plain is one of the most important maize-producing regions in China, accounting for 30% of its maize-growing area and total maize production of whole country [1]. Increasing plant density plays a significant role in increasing maize yield [2–5]. However, high plant density exacerbates interplant competition and decreases the amount of resources available per plant [4], which in cereal crops leads to a higher risk of leaf senescence [6] and a lower rate of post-anthesis net photosynthesis [5]. Earlier leaf senescence influences post-anthesis dry matter accumulation (DMA) [7–9], eventually decrease maize yield [10–14]. Therefore, great effort should be made to improve photosynthetic area post-anthesis and photosynthetic capacity to further increase DMA and yield maize.

Maize yield also depends on optimized root system [15], which is a bridge connecting the soil, root function, and yield [16]. Increasing the root distribution in deeper soil is conducive to yield improvement, especially at high plant density [17]. However, root growth is greatly restricted by the hard plough pan, a byproduct of the intensive use of mechanization from sowing to harvest, which deteriorates the physical condition of the soil [18–20]. In the Huang-Huai-Hai Plain, the average soil bulk density is 1.37 g cm^−3^ at a depth of 5–10 cm, and 1.51 g cm^−3^ in the soil plough pan, which is higher than the optimal soil bulk density (1.1–1.3g cm^−3^) for maize growth [21]. Soil bulk density affects maize root dry mass in the 20-60cm soil layer [22]. In soil with a higher soil bulk density, water and nutrient uptake as well as air and water infiltration are limited and the roots are poorly able to penetrate the soil [16, 23–25]. The higher soil compaction leads to a higher concentration of the root system in the upper soil layer and a reduction in the root distribution in deeper layers [16, 26]. Large proportion of roots distributed in the upper soil profile was likely to limit root extension and reduce the grain yield [27, 28]. Thus, how to improve soil quality is a main way to increase yield [29].

Subsoiling can effectively break up soil plough pan, reduce soil bulk density, and thereby promote root extension into deep soil layers [18, 30–31]. Subsoiling has also been shown to markedly improve the tilled layer [32] and rhizosphere microorganisms, microbial diversity, and soil water—storage capacity, thus establishing ideal conditions for root development and effectively delaying plant senescence [33–34]. Subsoiling increases grain yield and also makes it possible to increase planting density by 1.08–5.21% [35].

Coordination of the root system and canopy structure is essential to increase maize yield, however, the physiological processes affected by soil tillage and plant density is poorly understood. In this study, our objectives were to: (1) assess the effects of subsoiling on soil compactness, root system distribution, and root activity; (2) determine changes in DMA, leaf area, grain filling, and photosynthetic rate; (3) and investigate physiological processes that subsoiling regulates the post-anthesis aboveground DMA and yield.

## Materials and methods

### Site description

**Fig 1:**
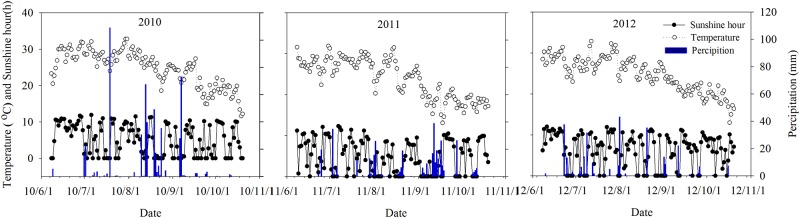
Changes in temperature, hours of sunshine, and precipitation during the maize growth period in 2010, 2011, and 2012 at the Xinxiang experimental station.

Field experiments were conducted from 2010 to 2012 at the Xinxiang Experimental Station of the Chinese Academy of Agriculture Sciences, Henan Province, China (35°11′30″N, 113°48′E). The cropping pattern of this area is largely a winter-wheat/summer-maize double-cropping system, with rotary tillage for wheat and no-till farming for maize, with the latter crop grown almost entirely under irrigated conditions. The annual mean temperature, hours of sunshine, and amount of precipitation in the study area are shown in Fig 1. The soil texture at the site is clay loam (ISSS Classification, International Soil Science Society), with 12.6 g organic material kg^−1^, 61.2 mg available N kg^−1^, 16.2 mg available P kg^−1^, 110.0 mg available K kg^−1^, and a pH of 8.21.

### Experimental design and field management

The maize hybrid ZD958 was chosen for this study, as it is the most widely grown cultivar in the Huang-Huai-Hai Plain and has a high yield, is multi-resistant, and is highly adaptable. Treatments consisted of planting patterns with wide (0.8 m) and narrow inter-row spacing (0.4 m); three different plant densities: low (LD, 45000 plants ha^−1^), medium (MD, 67500 plants ha^−1^), and high (HD, 90000 plants ha^−1^); and two soil tillage practices: no-tillage (CK), in which the crop residues after wheat harvest were flattened and remained on the soil surface and the only soil disturbance was during planting and fertilizer application, and subsoiling (SS), in which the soil was completely inverted and the crop residue was buried to the depth of 30 cm using a subsoiler (Hehuinong Machine Co. Ltd., Beijing, China).

The experiment was carried out according to a split-plot design: soil tillage practices were considered the main factors and the planting densities were sub-plot factors, with three randomized replicates. The experimental plots were 12 m long and 6.0 m wide and consisted of 10 rows. All plots received the same amount of water and nutrients. Before sowing, the plots were finely prepared, irrigated, and basal fertilizer was applied at a rate of 225 kg N ha^−1^, 173 kg P_2_O_5_ ha^−1^, and 150 kg K_2_O ha^−1^. In addition, N fertilizer (138 kg N ha^−1^) was applied at twelfth leaf stage. The amount of fertilizer applied was based on the existing levels of N, P, and K, as determined from soil tests, to ensure that there were no nutrient deficiencies. The plots were irrigated as needed from plant emergence to plant maturity to prevent water deficits. They were maintained free of weeds by herbicide application as needed. No obvious water stress or pest damage was observed during the growing seasons. The sowing dates were 8 June 2010, 14 June 2011, and 17 June 2012; the anthesis dates were 5 August 2010, 8 August 2011, and 11 August 2012; and the harvesting dates were 5 October 2010, 13 October 2011, and 19 October 2012.

Data from 2010 to 2012 were analyzed. Total precipitation during the growing season was 536.6 mm in 2010, 366.8 mm in 2011, and 278.8 mm in 2012. The total number of hours of sunshine was 630.3, 553.5, and 745.5, respectively. Thermal time computations used mean daily air temperature and a base temperature of 10°C. The total optimum temperature for maize growth was 1887.5°C, 1717.8°C, and 1825.0°C (Fig 1).

### Plant sampling

In each plot, three adjacent plants from the same inside row were cut manually at the ground level to determine the aboveground DMA 12, 31, 49, 68, and 104 days after emergence in 2010; 16, 34, 45, 75, and 110 days in 2011; and 20, 37, 50, 80, and 119 days in 2012. The harvested plants were heated initially at 105°C for 30 min and oven-dried at 75°C to a constant weight before weighing. Pre-anthesis and total DMA was determined at anthesis and harvesting stages. The post-anthesis DMA was calculated using the following formula:

$$\text{Post-anthesis DMA }({\text{t ha}}^{-1})\;=\text{ total DMA }-\text{ pre-anthesis DMA}$$ (1)

At harvest, a bordered area of 19.2 m^2^ (8.0 m × 2.4 m) in each plot was harvested by hand to determine the grain yield. The grain yield and kernel weight were adjusted to 14% moisture content. The 1000-kernel weight and the kernel number per ear were recorded.

### Leaf area index

The area of each fresh leaf from the sampled plants was determined immediately after harvesting (McKee, 1964). The leaf area index (LAI) was calculated as the leaf area × plant density (number of plants per m^2^).

### Grain development sampling

At least 50 representative plants were tagged in each plot 10 days before silking (the first silk visible on the apical ear). The silking date of the apical ear was recorded for each tagged plant, when the silks emerged in 50% of the plants in a plot. Three tagged ears of every plot were randomly selected plants were harvested at 5, 10, 15, 20, 25, 35, 45, 55, and 65 days after anthesis [36] and 100 grains were cut from the middle of each sampled ear [37]. The dry weight of 100 grains for each ear was measured after drying to a constant weight in a forced air oven at 80°C. The dynamics of grain weight during grain filling followed Richards’ growth equation:

$$\text{W}=\text{A }{\left(1+{\text{Be}}^{-\text{Ct}}\right)}^{-1/\text{D}}$$ (2)

where W is the grain weight, A is the ultimate grain weight, t is the day after pollination, and B, C, and D are coefficients determined by regression [38]. The data of the final grain weight was W_0_ = a, the maximum grain-filling rate was D_max_ = (lnB-lnD)/C, the grain weight at the maximum grain filling rate was W_max_ = A(D+1)^-1/D^, the maximum grain-filling rate was G_max_ = (CW_max_/D) (1-(W_max_/A)^D^), the mean grain-filling rate was G_mean_ = AC/2(N+2), the initial grain-filling potential was R_0_ = C/D, and the effect grain-filling duration was P = 2(D+2)/C.

### Leaf net photosynthetic rate and chlorophyll fluorescence

The ear leaf net photosynthetic rate (P_n_) was measured on sunny, windless days from 9:30 to 14:00 using the LI-6400 portable photosynthesis system (LI-COR Inc., Lincoln, NE, USA) equipped with an LED leaf chamber. Each leaf was measured in triplicate for each block. The photo flux density was controlled at 2000 μmol m^−2^s^−1^, and the air flow at 500 μmols^−1^. The CO_2_ concentration of the intake air was maintained at 350 μmol mol^−1^ [39–41]. The Genty’s method [42] was used to measure ear leaf chlorophyll fluorescence during anthesis and 20 and 69 days thereafter using the FMS portable modulated fluorometer (Lufthansa Instruments Inc., UK).

### Root and soil sampling

According to Wang’s methods, three plant roots were sampled per plot at anthesis using 10 × 10 × 10 small cubic sampling methods [15]. Plant was as the center of the soil samples with line spacing of 50cm (narrow-line spacing of 20cm, and wide-line spacing of 30cm), row spacing of 37cm in D1, 24.5cm in D2 and 18.5cm in D3, and depth of 0–10, 10–20, 20–30, 30–40, and 40-50cm, respectively. All of the visible roots in each soil block were harvested. Soil bulk density was measured according to the cutting-ring method as soil dry weight (g)/cutting-ring volume (cm^3^). Measurements were made at five soil depths (0–10, 10–20, 20–30, 30–40, 40–50 cm) in the anthesis stage. The measurements were repeated three times.

### Root bleeding sap collection

In each plot, three adjacent plants from the same inside row were cut manually at the 10 cm above the ground level to determine the root bleeding sap amount. The remnant stem incision was washed with deionized water. Stem diameter was measured using Vernier calipers, calculating the area of the cut plane. Root bleeding sap was collected from 6:00 pm until 6:00 am the next day using 500 ml plastic bottles [43], on days 0, 10, 30, 40, and 50 post-anthesis.

### Statistical analysis

The data were prepared using Microsoft Excel 2003, and statistical analyses were performed using SPSS 11.0. Means were compared using Duncan’s new multiple range method test at a probability (P) level of 0.05. Grain yield, yield components, were subjected a three-way analysis of variance with year, tillage and planting density as fixed effects. Pn, Fv/Fm, ΦSPII, grain filling rate parameters, root bleeding amount, and density of root dry weight were subjected a two-way analysis of variance with tillage and planting density as fixed effects.

## Results

### Grain yield

Tillage had significant effects on the kernels per ear, 1000-kernel weight and yield. Density and year had significant effects on ear number per ha^-1^, kernels per ear, 1000-kernel weight and yield, respectively. The interaction between tillage and density, tillage and year, and density and year had significant effects on yield. However, the interaction of three factors (tillage, density, and year) had no significant effects on yield (Table 1). The highest grain yields were obtained from the SS treatment: HD in 2010 and 2012 and MD in 2011. Compared to the control, grain yields increased between 2010 and 2012 by 6.28%, 7.31%, and 10.51%, respectively, with HD, MD, and LD. With increasing planting density, the 1000-kernel weight in the SS treatment increased as well, by 4.09% (LD), 5.13% (MD), and 4.96% (HD) compared to the CK treatment. The number of kernels per plant was also higher in the SS vs. the CK plants (3.04%, 6.06%, and 8.56%, respectively). There were no significant differences in ear number in 2010 and 2012 whereas in 2011 it was significantly higher in the SS than in the CK treatment. The average increases in 1000-kernel weight, kernels per plant, and ear number were 4.73%, 5.89%, and 2.10%, indicating that the higher yield under the SS treatment was mainly due to the increases in kernels per plant and 1000-kernel weight and therefore that subsoiling mainly affected grain formation and filling after anthesis.

**Table 1:** Maize grain yield, ear numbers per ha, kernels per ear, 1000-kernel weight of maize, as an effect of different tillage practices and different planting density in 2010, 2011, and 2012.

Years	Density (10^4^ plants ha^−1^)	Tillage	Ear numbers (10^4^ ha^−1^)	Kernels per ear	1000-kernel weight (g)	Grain yield (kg ha^−1^)
2010	LD	CK	5.88 c	489.12 b	308.25 a	8627.03 e
		SS	6.00 c	513.11 a	310.33 a	8914.08 de
	MD	CK	7.44 b	412.65 d	292.29 c	9186.12 d
		SS	7.45 b	431.31 c	301.39 b	9514.70 c
	HD	CK	9.37 a	356.98 f	284.40 d	9876.28 b
		SS	9.37 a	381.62 e	295.95 bc	10462.53 a
2011	LD	CK	5.00 f	548.77 b	302.60 a	8822.56 c
		SS	5.40 e	578.55 a	308.14 a	9320.06 b
	MD	CK	6.59 d	471.09 d	273.95 c	9297.23 b
		SS	6.75 c	530.32 c	287.14 b	9976.91 a
	HD	CK	8.17 b	376.35 f	257.54 d	7880.92 d
		SS	8.51 a	428.40 e	270.93 c	9165.76 b
2012	LD	CK	6.35 d	542.03 a	323.54 c	10393.60 e
		SS	6.31 d	535.42 a	355.12 a	11419.58 cd
	MD	CK	7.33 c	495.59 b	312.93 c	11153.80 d
		SS	7.27 c	500.90 b	336.28 b	12350.44 b
	HD	CK	8.95 b	428.05 d	296.22 d	11765.15 c
		SS	9.27 a	449.28 c	312.86 c	13001.18 a
Source of variation						
Tillage (T)			0.054ns	0.000***	0.000***	0.000***
Density (D)			0.000***	0.000***	0.000***	0.000***
Year (Y)			0.000***	0.000***	0.000***	0.000***
Tillage × Density (T×D)			0.455ns	0.098ns	0.191ns	0.0127*
Year × Tillage (Y×T)			0.530ns	0.000***	0.022*	0.000***
Year × Density (Y× D)			0.000***	0.000***	0.000***	0.000***
Year × Tillage × Density (Y×T×D)			0.084ns	0.024*	0.019*	0.410ns

The mean values of the different treatments in the 3 years are shown. Different letters indicate statistically significant differences at the *P* <0.05 level (ANOVA and Duncan’s multiple range test; n = 3).

*significant at *P*<0.05,

** significant at *P*<0.01,

*** significant at *P*<0.001,

and ns, not significant, *P* ≥ 0.05.

LD: planting density of 45000 plants ha^-1^, MD: planting density of 67500 plants ha^-1^, HD: planting density of 90000 plants ha^-1^; SS: subsoiling; CK: no tillage.

### Dry matter accumulation

**Fig 2:**
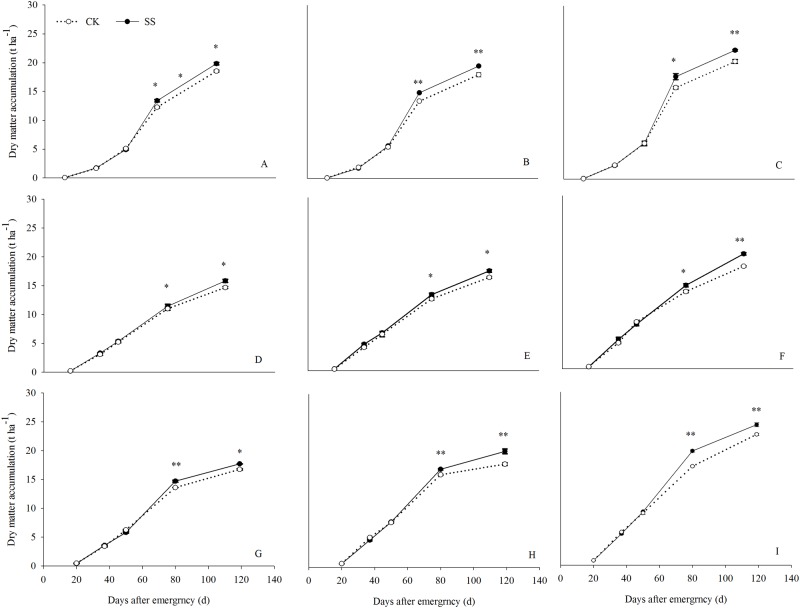
Effects of tillage management (SS, subsoiling; CK, no tillage) on maize dry matter in plots planted at density of 45000 plants ha^-1^, (LD), 67500 plants ha^-1^(MD), and 90000 plants ha^-1^ (HD) in each growth stage during 2010 (A), 2011 (D–F), and 2012 (G–I). Vertical bars represent the standard deviations of the means. * *P*< 0.05; **P< 0.01.

**Fig 3:**
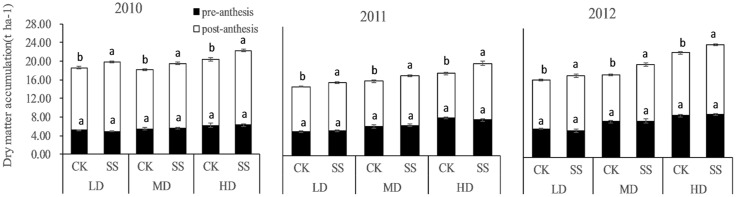
Dry matter accumulation pre- and post-anthesis in the control (no tillage, CK) and subsoiled (SS) crops from density of 45000 plants ha^-1^ (LD), 67500 plants ha^-1^(MD), and 90000 plants ha^-1^ (HD) in 2010 (A), 2011 (B) and 2012 (C). Vertical columns and bars represent the means and standard errors of three replicates, respectively. Different letters on the graph indicate significant differences at P< 0.05.

The pre-anthesis DMA did not significantly differ between the SS and CK plots during the three seasons (Figs 2 and 3), whereas during the grain-filling stage the DMA was significantly higher in the SS plots. During 2010, 2011, and 2012, the average total dry matter obtained from SS plots planted at high, medium, and low densities was 17.79, 18.97, and 22.21 t ha^−1^, respectively, which was 6.82%, 9.29%, and 9.51% higher than the values determined in the CK plots (16.65, 17.35, and 20.28 t ha^−1^). Further analyses showed that the post-anthesis DMA was greater for the SS than for the CK treatment for LD, MD, and HD, by 11.07%, 9.23%, and 13.44%, respectively, in 2010; 7.60%, 8.80%, and 26.49% in 2011; and 13.43%, 21.05%, and 10.73% in 2012 (Fig 3). The average increase in the SS treatment during the 3 years was 10.80%, 12.75%, and 15.82% for the LD, MD, and HD plots. In addition, total dry matter and post-anthesis dry matter were positively and significantly correlated (r = 0.8362***). These results suggest that subsoiling significantly improved both total and post-anthesis DMA.

### Grain filling characters

**Fig 4:**
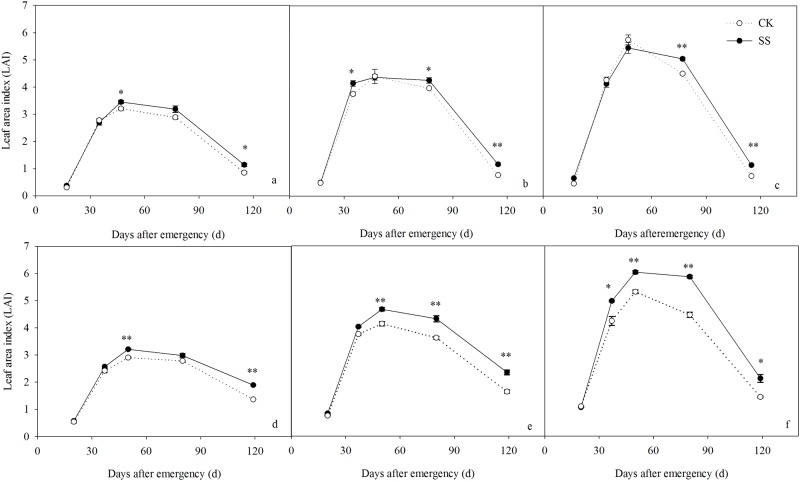
Grain-filling in crops from subsoiled (SS) and no-tilled (CK) plots planted at 45000 plants ha^-1^(LD, A), 67500 plants ha^-1^medium (MD, B), and 90000 plants ha^-1^(HD, C) during post-anthesis stage in 2012. Vertical bars are the standard deviations of the means. *P<0.05; **P<0.01, ***P<0.001.

Subsoiling had a marked influence on the 100-grain weight (Fig 4). The difference appeared earlier in the HD than in the MD and LD treatments. The grain-filling process was analyzed using Richards’ equation according to the four parameters associated with grain filling (Table 2): The W_0_, R_0_, T_max_, W_max_, P were affected significantly by the tillage practice × plant density interactions. At the lower plant density (D1), subsoiling mainly improve the T_max_ and W_max_. At higher plant density, the R_0_ and P under SS plots were higher than that CK plots. As the planting density increase, the positive effect of subsoiling on the G_mean_, G_max_, T_max_ and W_max_ were decreased, but the R_0_ and P were increased.

**Table 2:** Grain filling rate parameters in subsoiled (SS) and non-tilled (CK) plots in 2012.

Planting density	Soil tillage practice	Grain filling rate parameter						
		W_0_ (g 100 grain^-1^)	R_0_ (g 100 grain^-1^)	T_max_ (d)	W_max_ (g 100 grain^-1^)	P (d)	G_mean_ (g 100 grain^-1^d^-1^)	G_max_ (g 100 grain^-1^d^-1^)
LD	ck	34.72	1.11	20.69	13.30	44.16	1.16	0.79
	ss	38.93	0.86	22.61	15.16	47.20	1.22	0.83
MD	ck	32.76	0.25	23.16	14.57	40.78	1.19	0.80
	ss	36.39	0.39	22.87	15.05	43.85	1.22	0.83
HD	ck	30.95	0.23	23.93	14.07	41.41	1.11	0.75
	ss	36.91	1.22	22.49	14.03	49.30	1.10	0.75
Source of variation								
Tillage (T)		0.000***	0.004**	0.806ns	0.001**	0.000***	0.029*	0.034*
Density (D)		0.000***	0.000***	0.001***	0.013*	0.000***	0.000***	0.000***
Tillage × Density (T × D)		0.044*	0.000***	0.001***	0.003**	0.003**	0.284ns	0.142ns

W_0_, The final grain weight; R_0_, The initial grain-filling potential; T_max_, Maximum filling rate time; W_max_, Maximum filling rate of growth; P, Active filling phase; G_max_, Maximum filling rate; G_mean_, The mean grain-filling rate. LD: planting density of 45000 plants ha^-1^, MD: planting density of 67500 plants ha^-1^, HD: planting density of 90000 plants ha^-1^; SS: subsoiling; CK: no tillage.

*Significant at *P* < 0.05.

** Significant at *P* < 0.01.

*** Significant at *P* < 0.001.

ns, not significant, *P* ≥ 0.05

### Leaf area and Pn traits

**Fig 5:**
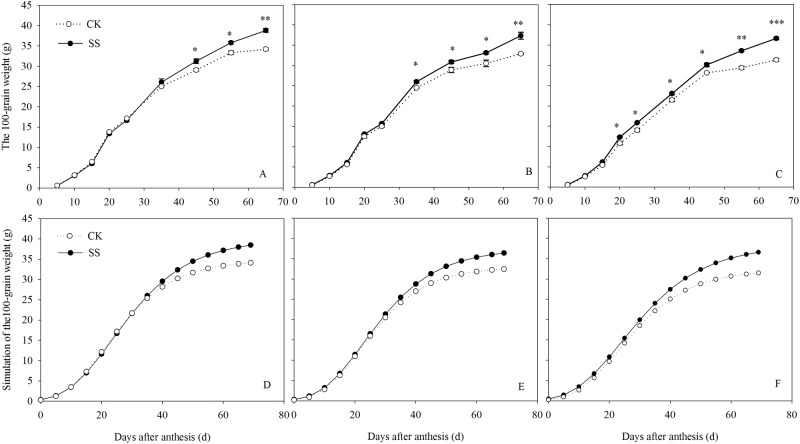
Leaf area index dynamics during the maize growth season in subsoiled (SS) and non-tilled (CK) plots planted at density of 45000 plants ha^-1^ (LD), 67500 plants ha^-1^(MD), and 90000 plants ha^-1^ (HD) in 2011 (A–C, respectively) and 2012 (D–F, respectively). Vertical bars are the standard deviations of the means. *P<0.05; **P<0.01.

The canopy production capacity is a function of the LAI. In our study, the LAI increased dramatically during the pre-anthesis stage, peaked at anthesis, and declined thereafter in all treatments (Fig 5). A positive effect of subsoiling on the LAI occurred mainly in the post-anthesis stage. In 2011, as the plant density increased from LD to HD, the LAI values of the SS-treated vs. CK-treated crops changed by 7.63%, -0.81%, and -5.22% at anthesis, 10.52%, 7.28%, and 12.14% in the mid-filling stage, and 34.10%, 51.26%, and 54.52% at harvest (Fig 5a–5c). In 2012, the LAI values increased consistently, by 10.46%, 12.81%, and 13.54% at anthesis; 7.08%, 19.30%, and 31.40% in the mid-filling stage; and 38.79%, 43.50%, and 47.39% at harvest (Fig 5d–5f). According to these results, subsoiling maintained a higher green leaf area and delayed leaf senescence such that the photosynthetic area of the canopy in the grain-filling stage was higher, with the most noticeable effects achieved in the HD plots.

**Fig 6:**
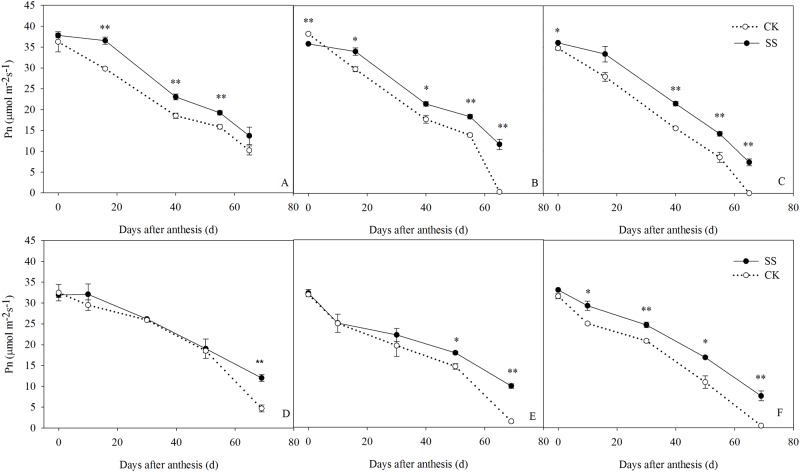
The net photosynthetic rate (Pn) of leaves post-anthesis in subsoiled (SS) and no-tilled (CK) plots planted at 45000 plants ha^-1^ (LD), 67500 plants ha^-1^ (MD), and 90000 plants ha^-1^ (HD) in 2011 (A–C, respectively) and 2012 (D–F, respectively). Vertical bars are the standard deviations of the means. *P<0.05; **P<0.01.

The net photosynthetic rate (Pn) decreased gradually after anthesis (Fig 6). Pn were affected significantly by the tillage × plant density interaction at 65 days post-anthesis in 2011, and at 69 days post-anthesis in 2012 (S1 Table). At the beginning of grain filling, there were no differences between the two tillage treatments, but in 2011 and 2012 the rate of decreasing was slower in the SS plots. The average Pn of the LD, MD, and HD plantings during these 2 years was 9.14%, 15.93%, and 25.60% higher in the SS than in the CK treatment. Thus, subsoiling did not enhance the Pn, but delayed the onset of leaf senescence.

**Fig 7:**
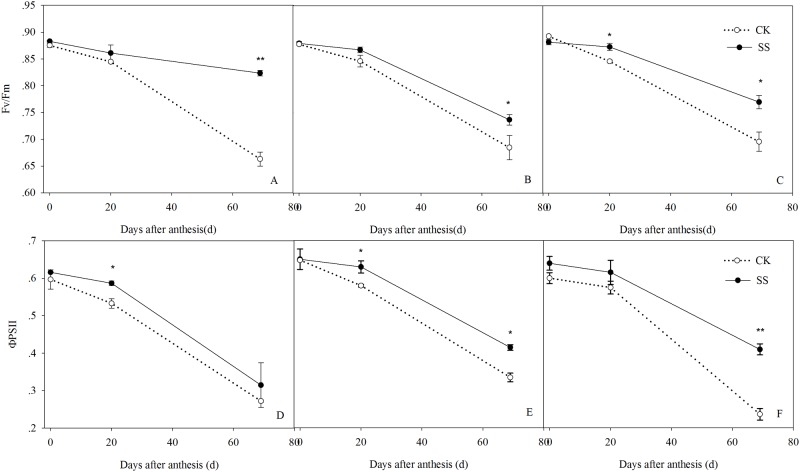
The F_v_/F_m_ (A–C) and ΦPSII (D–F) of the post-anthesis leaves from maize planted in plots of 45000 plants ha^-1^ (LD, A and D), 67500 plants ha^-1^ (MD, B and E), and 90000 plants ha^-1^ (HD, C and F) densities managed by subsoiling (SS) or no tillage (CK) in 2012. Vertical bars represent the standard deviations of the means. * *P*< 0.05; **P< 0.01.

Significant Tillage×Plant density interaction were observed for the maximum photochemical efficiency (F_v_/F_m_) at 0 and 69 days post-anthesis, and the maximum quantum efficiency of PSII (ΦPSII) at 60 days post-anthesis (S2 Table). The F_v_/F_m_ and ΦPSII decreased post-anthesis (Fig 7). The average F_v_/F_m_ of the SS crops planted at low, medium, and high densities was 0.86, 0.83, and 0.84, which was 7.42%, 3.10%, and 3.70% higher than the values determined in the CK treatment. Subsoiling also enhanced ΦPSII, by 8.26%, 8.50%, and 17%, respectively.

### Root traits and function

The tillage practice × plant density interaction effects for the density of root dry weight at 20-30cm soil profile were significant (Table 3). Subsoiling changed the root distribution in the 0–50 cm soil profile, as evidenced by the decrease in the root dry weight in the 0–10 cm soil profile and its dramatic increase in the 10–50 cm soil profile. At the 0–10 cm profile, the root dry weight in the SS plots decreased by 5.73% (LD), 6.81% (MD), and 4.83% (HD) compared to the CK plots whereas at the 10–50 cm soil depth, increases of 40.68%, 32.17%, and 20.14% were recorded. In the SS treatment, the average density of root dry weight at the 0–50 cm soil depth was 5.64%, 5.04%, and 1.82% greater in the LD, MD, and HD plots of the SS vs. the CK treatments. These results indicate that subsoiling significantly affected the depth distribution of the roots by promoting root penetration to the deep soil and reducing the pressure of root crowding at the soil surface, but without a significant effect on total root dry weight.

**Table 3:** Density of root dry weight (g m^-3^) at a soil depth of 0–50 cm soil depth during the anthesis stage of subsoiled (SS) vs. non-tilled (CK) plots in 2012.

Planting density	Treatment	Soil depth (cm)					
		0–10	10–20	20–30	30–40	40–50	0–50
LD	CK	558.13 a	87.72 b	38.75 b	27.40 b	27.31 b	147.86
	SS	526.15 b	116.85 a	55.57 a	41.50 a	40.97 a	156.21
	±Δ(%)	−5.73	33.21	43.41	51.46	50.02	5.64
MD	CK	534.89 a	124.69 b	43.93 a	33.57 b	31.53 b	153.72
	SS	498.47 b	151.11 a	69.14 b	46.99 a	41.67 a	161.48
	±Δ(%)	−6.81	21.19	57.39	39.98	32.16	5.04
HD	CK	509.20 a	92.78 b	39.23 b	30.20 b	22.59 b	138.80
	SS	484.59 b	112.33 a	43.33 a	34.27 a	32.10 a	141.32
	±Δ(%)	−4.83	21.07	10.44	13.47	42.10	1.82
ANOVA	Tillage (T)	0.000***	0.000***	0.000***	0.001**	0.000**	0.017*
	Density (D)	0.000***	0.000***	0.000***	0.058ns	0.003***	0.000***
	Tillage × Density (T ×D)	0.730ns	0.683ns	0.003**	0.231ns	0.608ns	0.527ns

LD: planting density of 45000 plants ha^-1^, MD: planting density of 67500 plants ha^-1^, HD: planting density of 90000 plants ha^-1^; SS: subsoiling; CK: no tillage. Different letters indicate statistically significant differences at the p<0.05 level (ANOVA and Duncan’s multiple range test; n = 3).

*Significant at *P* < 0.05.

** Significant at *P* < 0.01.

*** Significant at *P* < 0.001.

ns, not significant, *P* ≥ 0.05

**Fig 8:**
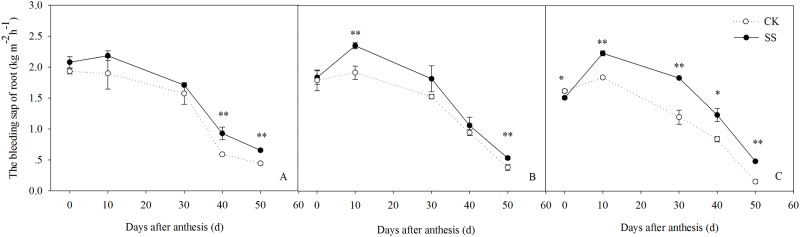
The amount of root bleeding sap determined in plants from plots planted at 45000 plants ha^-1^ (A), 67500 plants ha^-1^ (B), and 90000 plants ha^-1^ (C) at during post-anthesis under subsoiling (SS) and non-tillage (CK) conditions in 2012. Vertical bars represent the standard deviations of the means. * *P*< 0.05; **P< 0.01.

Interaction effects between tillage practice and plant density on the root bleeding sap amount were significant at 50 days after anthesis (S3 Table). The root bleeding sap amount was significantly higher in the SS plots than in the CK plots at each developmental stage during post-anthesis (Fig 8). The mean root bleeding sap amount of the SS crops was 1.51, 1.52, and 1.46 kg m^−2^h^−1^ under the LD, MD, and HD conditions, respectively, which was 17.41%, 15.82%, and 20.91% higher than the CK treatment values. These results suggest that subsoiling effectively promotes root growth and delays root senescence.

### Soil characteristic improvement

**Fig 9:**
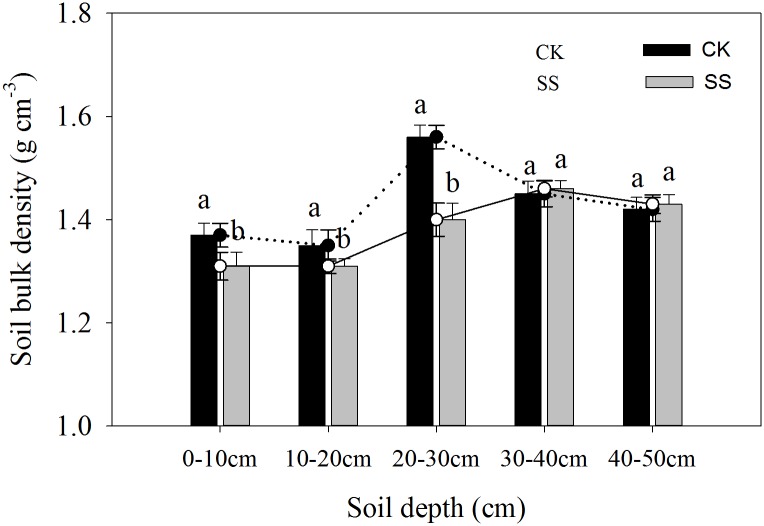
Soil bulk at depth intervals between 0 and 50 cm during the maize anthesis stage in plots managed by subsoiling (SS) or no tillage (CK) in 2012. Error bars represent the standard error of the mean. Different letters above the error bars indicate significant differences at the P<0.05 level (ANOVA and Duncan’s multiple range test; n = 3).

Soil bulk density was clearly modified by soil tillage (Fig 9). Subsoiling mainly broke up compaction of the 0–30 cm soil layer, especially the 20–30 cm layer. Soil bulk densities in the 0–10, 10–20, and 20–30 cm soil profile layers (0.06, 0.04, and 0.16 g cm^−3^, respectively) but not in the 30–50 cm soil layer were significantly lower in the SS plots than in the CK plots.

## Discussion

### Effects of subsoiling on topsoil characteristics and roots

Maize growth and yield are closely related to a strong root system. Tillage practices change the temporal and spatial structure of the soil to affect the spatial distribution of the roots [44]. The plough pan, affecting the 15–30 cm soil layer, strongly restricts root growth and accelerates root senescence. Optimal tillage practices provide a good topsoil layer, one that promotes maize root growth through the exchange of soil water, fertilizer, gas, and heat with the external environment [45]. Subsoiling effectively breaks up the plough plan, thereby improving soil properties by generating a less restricted physical environment that stimulates root growth and increases the proportions of roots in the deeper soil [46]. Our data showed that subsoiling effectively decreased soil bulk density in the 0–30 cm soil layer, by 5.87% compared to the non-tilled (CK) plots. The soil bulk density in the 20–30 cm soil layer decreased by 10.26% in the SS vs. CK treatment, a consequence of the subsoiler’s working depth of 30 cm. Subsoiling altered the root distribution in the different soil layers. Thus, in the SS treatment, the root dry weight in the 0–10 cm soil layer was lower but in the deeper (10–50 cm) soil layer it was higher than in the CK treatment. The proportion of roots at 0–10 cm was 72.82% in the CK plots vs. 68.89% in the SS plots; the corresponding values were 13.82% and 16.52% in the 10–20 cm layer, 5.53% and 7.27% in the 20–30 cm layer, 4.14% and 5.33% in the 30–40 cm layer, and 3.68% and 4.98% in the 40–50 cm layer. We also found the interaction effects of the tillage and plant density on the root traits were mainly occurred at the 20-30cm soil profile, and the increase rate was highest at the MD plant density under SS treatment. These results demonstrate that subsoiling reduces the root distribution at the surface soil and promotes root growth in the deeper soil, thus alleviating root crowding and competition in the topsoil [16] while promoting root growth in the deeper soil layer to improve soil water and nutrient utilization. These findings are consistent with those of previous studies [44, 47].

Maintaining higher root activity is an essential cultivation technique to increase maize yield. Our results showed that the amount of root bleeding sap, a standard measure of root activity, was higher in the SS than in the CK plots. The effects of subsoiling on root vigor was more apparent at the higher planting density, as evidenced by a mean increase in 17.41% in the LD treatment vs. 20.91% in the HD treatment. Previous studies have shown that enhancing the root distribution in the deeper soil layer improves root activity and delays root senescence [48], presumably because of the more stable root environment, which enhances the stress resistance of maize. In our study, by disrupting the plough pan located in the 20–30 cm soil layer, subsoiling allowed an extension of the root system within the 10–50 cm layer. This ensured water and nutrient uptake by the root system in deep soil. Thus, in summary, subsoiling creates an environment that favors root growth and delays root senescence, resulting in higher root activity at the late grain-filling period.

### Effects of subsoiling on post-anthesis photosynthesis and canopy leaf photosynthetic function

A strong root system plays an important role in supporting aboveground crop growth, and optimum canopy function is a key factor in achieving a higher yield. As an indicator of canopy architecture at different growth periods, Leaf area index (LAI) is an important indicator of the canopy architecture at different growth period, and longer green leaf area duration at post-anthesis period is one of several morpho-physiological traits associated with improvement of maize yield [49]. In this study, the post-anthesis LAI was significantly higher in the SS than in the CK plots. This suggests that subsoiling supports a higher green leaf area to provide a larger source of photosynthate post-anthesis, such that high levels of leaf photosynthesis are possible in the late grain-filling stage and DMA is increased accordingly. Photosynthesis, as the basis of biomass production and yield [39, 50], reflects the extent of leaf senescence [51]. We found a higher Pn in the SS than in the CK plots, particularly in the late grain-filling stages. As the planting increased from LD to HD, the average post-anthesis Pn in the SS plants was 13.44–27.59% higher in the SS plots; the F_v_/F_m_ and ΦPSII of the SS crops were also higher. Previous studies have shown that the higher photochemical efficiency is essential for efficient photosynthetic functioning [52] and the rapid decreasing of photochemical activity is an important reason for photosynthetic function sharp declines [53]. Our results suggest that SS preserves a higher Pn, F_v_/F_m_, and ΦPSII to maintain a higher green leaf area, prolong the duration of leaf photosynthesis post-anthesis, and effectively delay leaf senescence such that the leaf assimilation capacity is higher.

### Effects of subsoiling on DMA and yield

The main contributor to grain yield is DMA [54]. Our results showed that SS improves both total and post-anthesis DMA but not pre-anthesis DMA. The average increase in post anthesis DMA in the SS treatment during the 3 years was 13.12%. Relative to the CK treatment, subsoiling clearly maintained higher LAI, Pn, F_v_/F_m_, and ΦPII values and delayed leaf senescence during the grain-filling period, probably due to the deeper root distribution and the higher root activity in the SS plots. The higher LAI and green leaf photosynthetic activity enabled a high rate of DMA to support maize grain growth. Thus, SS improved canopy photosynthetic ability to achieve a higher post-anthesis DMA. A correlation analysis between pre-anthesis, post-anthesis, total DMA, and yield showed that post-anthesis DMA was significantly and positively correlated with total DMA and grain yield, as previously reported [55–56]. The higher post-anthesis DMA was the basis for the higher grain yield in the SS treatment.

Tillage practices change soil compaction, which affects root growth and distribution, and ultimately yield [18]. As shown in our experiment, subsoiling significantly increased maize production in the LD, MD, and HD plots, by 4.80%, 7.20%, and 8.92%, respectively. A yield components analysis for the 3 years showed consistently higher grain numbers and 1000-grain weights, both of which are established during the grain-filling stage [57], in the SS than in the CK plots, with average increases of 5.82% and 4.78%, respectively. In addition, the higher DMA under SS treatment resulted in a higher grain number and grain weight and significantly increased grain filling. SS could increase the initial grain filling potential and extend the effective grain filling duration. which is associated with significantly higher grain weight. This is because the SS improve the canopy function and delay the leaf senescence to grain filling. Especially, at higher planting density, the effect of on the R_0_ and P was more obvious. Taken together, these findings indicate that subsoiling increases maize yield by improving both the physical properties of the soil [58] and the level of photosynthetic assimilation, which together favor grain growth. The positive effect of subsoiling on the dry matter accumulation, LAI, Photosynthetic characteristics was increased with the plant density increasing, and the effect of subsoiling was most significant at the plant density of 90000 plant ha-^1^. In addition, the effects of tillage and density on yield were different in different years. In 2010 and 2012 year, the yield of SS plots was largest at HD plant density, and MD plant density was followed. In 2011 year, subsoiling produced a higher yield as the plant density increased from LD to MD. As the plant density increased from MD to HD, no further improvement in grain yield was achieved. In 2011, because of fewer ears, the HD plots did not produce a higher yield, perhaps due to the 27.62% increase in rainfall and the 45.43% decrease in the amount of sunshine compared to 2012. Also in 2011, most of the increased rainfall occurred at post-anthesis, and in combination with the reduction in the amount of sunlight, increased the mutual shading of plants under the higher population density. Previous research also showed that high temperatures and rainfall and low solar radiation had a negative impact on maize yield during the flowering and grain filling periods [59]. Nonetheless, our results are consistent with a positive effect of subsoiling on maize yield. Hence, subsoiling not only can improve the soil characteristic and root traits, but also can optimize the canopy performance in terms of leaf area, dry matter accumulation, Pn traits and higher yield.

## Conclusion

Our results demonstrate that subsoiling is an effective tillage management measure to improve summer maize yield, particularly under higher planting densities. Subsoiling improves the physical properties of soil, reduces root density in surface soil, and increases deep root growth, which alleviates root crowding in the upper soil layer and enhances water and nutrient absorption in the deeper soil layer. They delay leaf senescence after anthesis and maintain greater green leaf area and photosynthetic capacity, which promotes post-anthesis DMA and grain filling, and eventually increases grain yields by 6.21–10.09% (S1 Fig).

## Supporting information

S1 Fig The subsoiling adjustment process in the maize canopy and in the plough layer. (TIF)

S1 Table The two-way ANOVA by tillage and plant density for maize leaf Pn at post-anthesis. (DOCX)

S2 Table The two-way ANOVA by tillage and plant density for maize leaf fluorescence parameters at post-anthesis. (DOCX)

S3 Table The two-way ANOVA by tillage and plant density for root bleeding sap amount at post-anthesis. (DOCX)
