# Supplementary figures and images for: Subsoiling practices change root distribution and increase post-anthesis dry matter accumulation and yield in summer maize
Source: PLoS One. 2017 Apr 6;12(4):e0174952. doi: 10.1371/journal.pone.0174952 (PMC5383055; doi:10.1371/journal.pone.0174952)

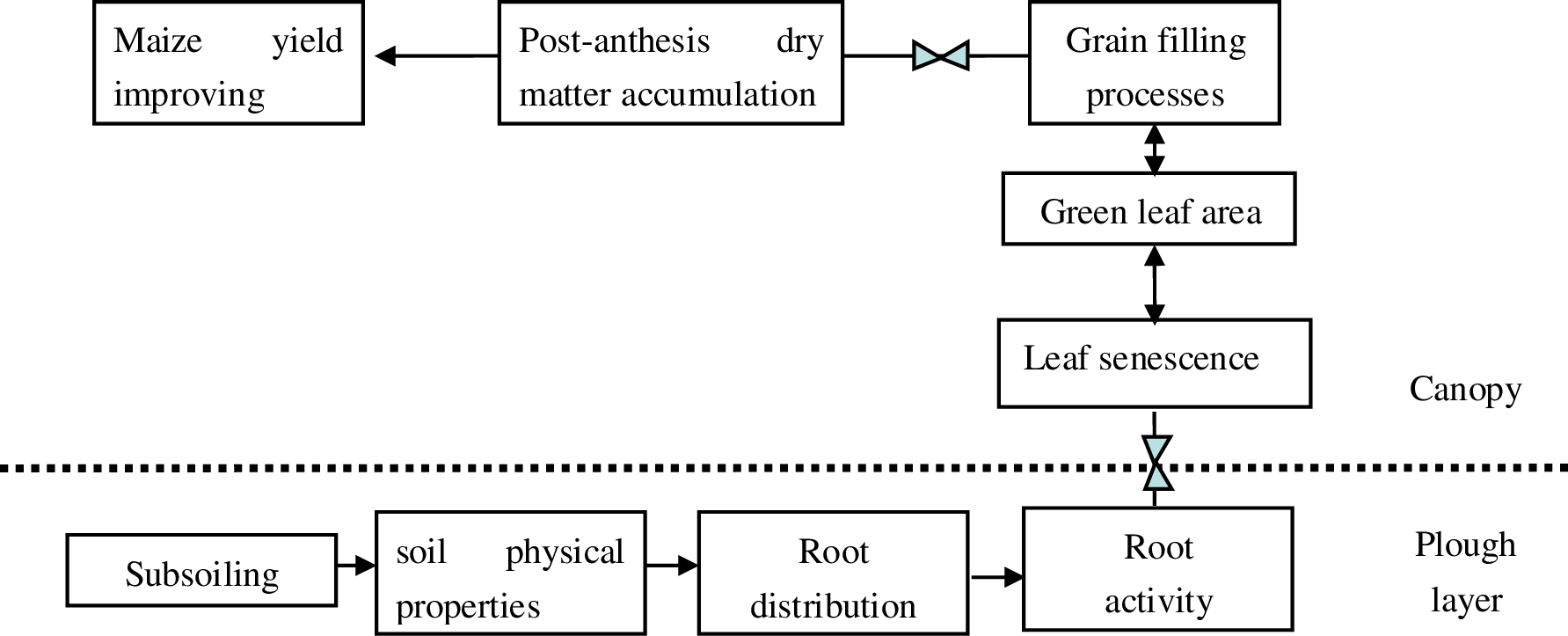

Supplement: S1 Fig — (TIF) [file pone.0174952.s001.tif]
