# Supplementary material for: Subsoiling practices change root distribution and increase post-anthesis dry matter accumulation and yield in summer maize
Source: PLoS One. 2017 Apr 6;12(4):e0174952. doi: 10.1371/journal.pone.0174952 (PMC5383055; doi:10.1371/journal.pone.0174952)
Supplement: S1 Table — (DOCX) [file pone.0174952.s002.docx]

**S1 Table. The two-way ANOVA by tillage and plant density for maize leaf Pn at post-anthesis**

| Treatment | Days after anthesis | | | | | | | | | |
| --- | --- | --- | --- | --- | --- | --- | --- | --- | --- | --- |
|  | 2011 | | | | | 2012 | | | | |
|  | 0 d | 16 d | 40 d | 55 d | 65 d | 0 d | 10 d | 30 d | 50 d | 69 d |
| Tillage (T) | 0.868 ns | 0.000 *** | 0.000 *** | 0.000*** | 0.000 *** | 0.782 ns | 0.079 ns | 0.022 * | 0.006 ** | 0.000 *** |
| Plant density (D) | 0.248 ns | 0.100 ns | 0.013 * | 0.000 *** | 0.000*** | 0.956 ns | 0.007 ** | 0.001** | 0.005 ** | 0.000 *** |
| Tillage ⅹ Density (T ⅹ D) | 0.147 ns | 0.512 ns | 1.599 ns | 1.441 ns | 0.019* | 0,492 ns | 0.380 ns | 0.252 ns | 0.116 ns | 0.005 ** |

* The differences are significant at p<0.05 level; ** The differences are significant at p<0.01 level; *** The differences are significant at p<0.001 level; ns, Non-significant, p>0.05 level.
