# Supplementary material for: Subsoiling practices change root distribution and increase post-anthesis dry matter accumulation and yield in summer maize
Source: PLoS One. 2017 Apr 6;12(4):e0174952. doi: 10.1371/journal.pone.0174952 (PMC5383055; doi:10.1371/journal.pone.0174952)
Supplement: S2 Table — (DOCX) [file pone.0174952.s003.docx]

**Table2 The two-way ANOVA by tillage and plant density for maize leaf fluorescence parameters at post-anthesis**

| Treatment | Days after anthesis | | | | | |
| --- | --- | --- | --- | --- | --- | --- |
|  | FV/FM | | | ΦPSⅡ | | |
|  | 0 d | 20 d | 69 d | 0 d | 20 d | 69 d |
| Tillage (T) | 0.822 ns | 0.007 ** | 0.000 ** | 0.101 ns | 0.000 ** | 0.000 ** |
| Density (D) | 0.020 * | 0.182 ** | 0.033 * | 0.032 ** | 0.008 ** | 0.000 ** |
| Tillgage ⅹ Density (TⅹD) | 0.024 * | 0.890 ns | 0.001 ** | 0.443 ns | 0.841 ns | 0.004 ** |

* The differences are significant at p<0.05 level; ** The differences are significant at p<0.01 level; *** The differences are significant at p<0.001 level; ns, Non-significant, p>0.05 level.
