# Supplementary material for: Subsoiling practices change root distribution and increase post-anthesis dry matter accumulation and yield in summer maize
Source: PLoS One. 2017 Apr 6;12(4):e0174952. doi: 10.1371/journal.pone.0174952 (PMC5383055; doi:10.1371/journal.pone.0174952)
Supplement: S3 Table — (DOCX) [file pone.0174952.s004.docx]

**S3 Table. The two-way ANOVA by tillage and plant density for root bleeding sap amount at post-anthesis**

| Treatment | Days after anthesis (d) | | | | |
| --- | --- | --- | --- | --- | --- |
|  | 0d | 10d | 30d | 40d | 50d |
| Tillage (T) | 0.709 ns | 0.000 *** | 0.001 ** | 0.000 *** | 0.000 *** |
| Density (D) | 0.001 ** | 0.458 ns | 0.284 ns | 0.003 ** | 0.000 *** |
| Tillage ⅹ Density (TⅹD) | 0.358 ns | 0.690 ns | 0.077 ns | 0.147 ns | 0.001 *** |

* The differences are significant at p<0.05 level; ** The differences are significant at p<0.01 level; *** The differences are significant at p<0.001 level; ns, Non-significant, p>0.05 level.
